# Expression of a β-Mannosidase from *Paenibacillus polymyxa* A-8 in *Escherichia coli* and Characterization of the Recombinant Enzyme

**DOI:** 10.1371/journal.pone.0111622

**Published:** 2014-11-25

**Authors:** Xi Bai, Hong Hu, Huaping Chen, Quanbin Wei, Zeshen Yang, Qianming Huang

**Affiliations:** 1 College of Science, Sichuan Agricultural University, Yaan, Sichuan 625000, P. R. China; 2 College of Animal Science, Anhui Science and Technology University, Fengyang, Anhui 233100, P. R. China; 3 Liang Shan Zhong Ze New Technology Development Co., Ltd, Xichang, Sichuan 615000, P. R. China; University of Alberta, Canada

## Abstract

*Paenibacillus polymyxa* A-8, which secretes β-mannosidase, was isolated from the soil sample under a pine tree located in the “Laoban” mountain region of Sichuan, China. The β-mannosidase gene (*MANB*) was isolated from *P. polymyxa* A-8, using primers according to the complete genome. The *MANB* (2,550 bp) encoding 849 amino acid residues was expressed in *Escherichia coli*. The specific activities of β-mannosidase produced by *P. polymyxa* A-8 and *E. coli* pET30a-*MANB* were 12 nkat/mg and 635 nkat/mg respectively. SDS-PAGE analysis indicated that the molecular mass of the recombinant MANB was approximately 96 kDa. The recombinant MANB was active between pH 7.0–8.5 with the maximum activity at pH 7.0. It had good pH stability and adaptability. The MANB had the optimal temperature of 35°C and was relatively stable at 35–40°C. In addition, the MANB activity was enhanced by K^+^, Ca^2+^, Mn^2+^, and Mg^2+^ and inhibited by Zn^2+^, Cu^2+^, and Hg^2+^.

## Introduction

Mannan is the second largest component of the hemicellulose and is distributed in plant cell walls [1]–[2]. It is classified into four subfamilies: galactoglucomannan, galactomannan, glucomannan and linear mannan. These saccharans contain β-1,4-mannose or α-1,6-mannose and mannose residues [3]. Due to the structural complexity and heterogeneity of plant mannan, its complete hydrolysis needs the synergistic action of mannanases, galactosidases and mannosidases. One of these important mannan-degrading enzymes is β-mannosidase [4]–[7].

β-Mannosidase (EC 3.2.1.25, MANB) catalyzes the hydrolysis of β-1,4-mannosidic bonds by releasing a single β-D-mannose unit from the nonreducing end of mannooligo saccharides [8]–[11]. There are many potential applications for mannosidases in industrial fields, such as increasing the quality of animal feed and food, bleaching of softwood pulp in paper industry, and producing the oligosaccharides for the medical industry [12]–[15].

The various microorganisms, including bacteria, archaea, fungi and actinomycetes, have been reported to secret β-mannosidases [16]–[19]. However, the application of β-mannosidases is still not extensive in the animal feed industry due to its low production level. Therefore, the identification of a new mannosidase with high activity is very desirable. Recently, the exogenous expression of enzymes has attracted considerable interest in order to improve their production level. In addition, the pET vectors are the most effective plasmids for the expression of proteases in *Escherichia coli* under the control of the bacteriophage T7 promoter, which can greatly increase their production level [20]–[21].

In the present work, the bacterium *Paenibacillus polymyxa* A-8 was isolated from the soil sample under a pine tree located in the “Laoban” mountain region of Sichuan, China. The β-mannosidase gene (*MANB*) from *P. polymyxa* A-8 was cloned and expressed in *E. coli* with the vector pET30α. In addition, the biochemical properties of the recombinant MANB were fully determined.

## Materials and Methods

### Strains, media and plasmids

This experiment was approved by Sichuan Agricultural University. *P. polymyxa* was isolated from the soil under a pine tree located in the “Laoban” mountain region near Sichuan Agricultural University (Address: Yaan, Sichuan 625000 P. R. China). The *P. polymyxa* was cultivated at 30°C for 24 h in Konjac flour medium to induce β-mannosidase. *E. coli* BL21 was used to express the gene *MANB*. The pTG19-T (Generay, China) was the T-cloning plasmid. The expression plasmid, pET30a, was stored in our laboratory.

### The sequence analysis of 16S rRNA

The 16S rRNA of the isolated microorganism was obtained by polymerase chain reaction (PCR) with the universal primers 5′-AGAGTTTGATCCTGGCTCAG-3′ (forward) and 5′-TACCTTGTTACCACTT-3′ (reverse). The PCR reaction mixtures (25 µL) included approximately 10 ng of isolated strain DNA, 0.1 µM each primer, and 12.5 µl PCR premix (Tiangen, China). The thermal program was as follows: 6 min at 94°C: followed by 32 cycles of 94°C for 38 s, 59°C for 50 s, and 72°C for 1.5 min. The purified PCR products were ligated into the vector pTG19-T and sequenced by Generay.

### Cloning of the *P. polymyxa* A-8 *MANB* gene

Genomic DNA extracted from *P. polymyxa* A-8 was used as the template DNA for PCR. To amplify the *MANB* gene, both primers were designed according to complete genome of *P. polymyxa* M1 [22]. The forward primer was 5′-GCTGGTACCATTAATGTGATAGGGTTGG -ATG-3′ (MANBF, the *Kpn*I restriction site is underlined and the start codon was removed), and the reverse primer was 5′-ATTGCGGCCGCCTAACCTTGTATCCAATTGATCA-3′ (MANBR, the *Not*I restriction site is underlined). The PCR mixtures and program were similar to the above description. The resulting product was inserted into the pTG19-T vector.

### Sequence analysis

The sequence analysis of *MANB* from *P. polymyxa* A-8 was performed by the ClustalW program, DNAstar 7.1 software and the BLAST server in GeneBank.

### Expression of the *MANB* gene in *E. coli*

The vector pTG19-MANB was double-digested with *Kpn*I and *Not*I, and the isolated *MANB* fragment was ligated into the pET30a vector. Next, the resulting vector pET30a-MANB was transformed into *E. coli* BL21.

The single *E. coli* pET30a-MANB harboring *MANB* was cultivated in 50 mL fresh LB medium kanamycin (30 µg/ml) and grown at 37°C for 4 h. Then, isopropyl β-D-thiogalactopyranoside (IPTG) was added into the culture broth at a final concentration of 1 mM. After cultivation for an additional 4 h, the cells were collected and fully resuspended in 10 mL of 0.05 M Tris/HCl buffer (pH 7.0), followed by ultrasonication. After centrifugation, the supernate was stored at −70°C.

### SDS-PAGE

SDS–PAGE was performed on a 12% running gel and stained by Coomassie brilliant blue [23].

### Protein purification

The supernatants containing the crude recombinant MANB were purified by a 6× His-Tagged Protein Purification kit (Cwbiotech, China) according to the manuals. The purified recombinant protein was stored at −70°C for analysis.

### Enzyme activity assay

The MANB activity was determined according to the methods described by Fliedrováet et al. [19]. Standard assay mixtures contained 50 µl of 1.25 mM p-nitrophenyl-β-D-mannopyranoside (pNPM), 100 µl of 50 mM of the enzyme solution and 400 µl of 0.05 M Tris/HCl buffer (pH 7.0) at 45°C for 30 min. The reaction was stopped by the addition of 3 ml of 0.2 M sodium carbonate solution, and the release of pNP was measured at 410 nm. One unit of enzyme activity (U) was defined as the amount of enzyme that released 1 µmol of p-nitrophenol per minute under the assay conditions.

### The effect of pH, temperature and metal ions on the activity of the recombinant MANB

The optimal pH of MANB was examined at 35°C for 30 min by using various buffers of pH 6–9 (50 mM citrate phosphate, pH = 6; 50 mM Tris/HCl, pH = 7–9). The pH stability of MANB was tested by pre-incubating the enzyme in different buffers (pH 6–9) for 30 min without substrate before detecting its residual activity.

The optimum temperature of the recombinant enzyme was determined at 20 to 50°C and pH 7.0. To examine its temperature stability, the MANB was incubated at 35°C, 40°C, and 45°C, respectively, for 30 min. In addition, its thermostability was determined by incubating the recombinant MANB for various times (30, 60, 90 and 120 min) at different temperatures (35°C and 45°C), and its residual mannosidase activity was then detected under the standard conditions as described above.

The effect of different metal ions on the activity of recombinant MANB was examined in 50 mM Tris/HCl buffer (pH 7.0) which contained 10 mM of ZnCl_2_, KCl, FeCl_3_, CaCl_2_, MnCl_2_, MgCl_2_, CuCl_2_ or HgCl_2_. After the reaction, the mixture was centrifuged. Then the supernate was used to determine its activity. The mannosidase activity of the assay system in the absence of any additives was considered to be 100%.

## Results and Discussion

### Isolation and identification of *P. polymyxa* A-8

**Figure 1:**
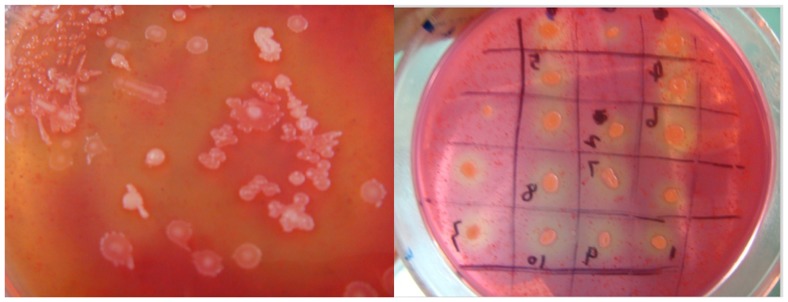
Bacterial isolates with zone of hydrolysis on the agar broth containing 0.4% congo red and 0.5% konjac powder.

According to the forming of clear zones around the colonies, the plate technique was used to isolate mannosidase microorganisms [24]. As shown in Figure 1, 10 colonies produced clear zones of hydrolysis in the agar broth. The results indicated that the specific MANB activity of strain A-8 was 12 nkat/mg, which was higher than the β-mannosidase activity in *Thermotoga neapolitana* 5068 reported by Duffaud et al. (1997) [25]. Therefore, the strain A-8 was chosen for further investigation.

**Figure 2:**
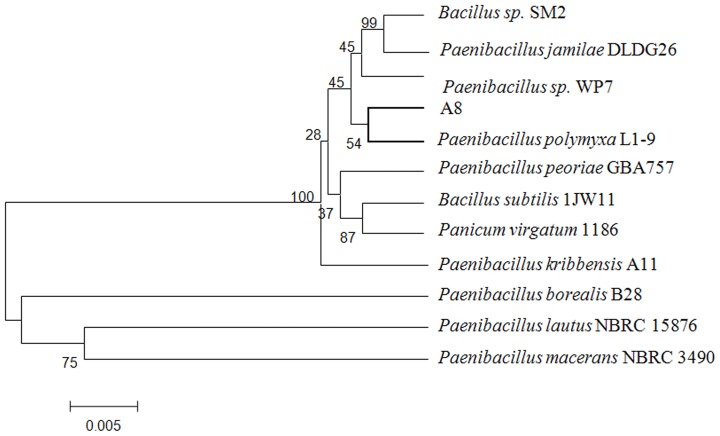
The phylogenetic tree of strain A-8 based on the sequence of 16S rRNA. The 16S rRNA of the strain A-8 sequence showed over 99% identity with the *P. polymyxa* L1-9.

Compared to the morphology, the microbial 16S rRNA sequence can more easily and accurately be used to classify a bacterium [26]. In the present study, the 16S rRNA of the strain A-8 (1459 bp) was amplified to identify its species. The GeneBank database and Mega 5.1 software were used to build a phylogenetic tree according to the alignment of the 16S rRNA sequence, and the results indicated that the A-8 strain was genetically more similar to *P. polymyxa* L1-9 than to the other microorganisms (Figure 2). Consequently, the A-8 strain was identified to be *P. polymyxa*.

### Cloning and sequencing of *MANB*

The targeted DNA fragment was amplified from the genomic DNA of *P. polymyxa* A-8 using the sequence-specific PCR primers MANBF and MANBR. The resulting fragment was 2550 bp, encoding 849 amino acid (aa) residues with an evaluated MW of 96.3 kDa. Use of the BLAST program by NCBI showed that the nucleotide sequence of *MANB* gene had 94%, 80% and 77% identity to the *Paenibacillus polymyxa* M1 genome (HE577054), *Arthroderma otae* CBS *MANB* (XM_002846187) and *Trichophyton verrucosum* HKI *MANB* (XM_003022716). The sequence for *MANB* was deposited in the GeneBank database under the accession number of KF576220.

**Figure 3:**
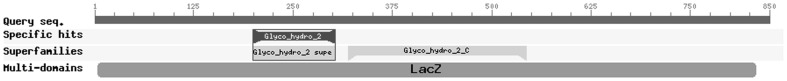
The putative domain of the protein belonged to GHs family 2 (Glyco_hydro_2).

β-Mannosidases are grouped into family 1, 2, and 5 of Glycosyl hydrolases (GHs) [8], [17], [27], and the amino acid sequence analysis indicated that the MANB of *P. polymyxa* A-8 belongs to the GHs family 2 (Figure 3). This family includes β-mannosidase, β-glucuronidase and β-galactosidase activities [17].

### Expression and purification of MANB in *E. coli*

**Figure 4:**
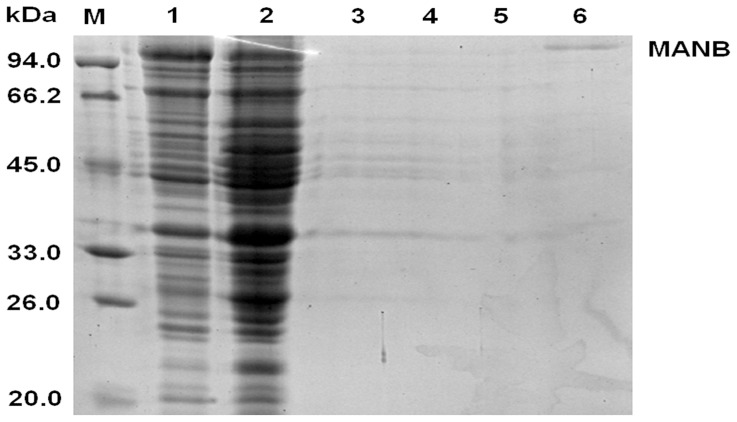
SDS-PAGE analysis. Lane M: protein MW markers; Lanes 1–2: IPTG induced *E. coli* pET30a-MANB; Lanes 3–5: Negative control; Lane 6: the purified MANB.

The gene which encodes MANB was expressed in *E. coli*. The recombinant *E. coli* pET30a-MANB was cultivated in LB broth and induced by IPTG. SDS-PAGE analysis revealed that the MW of the expressed protein was apprx. 96 kDa corresponding with that evaluated from the amino acid sequence (Figure 4). The specific activity of the cultured containing the crude recombinant MANB was 635 nkat/mg, which was higher than the MANB activity secreted by *P. polymyxa* A-8 and many other bacteria [5], [25].

The crude protein containing recombinant MANB was purified by a 6× His-Tagged Protein Purification kit, and a single band of approx. 96 kDa was presented on SDS-PAGE gel (Figure 4).

### The properties of MANB

**Figure 5:**
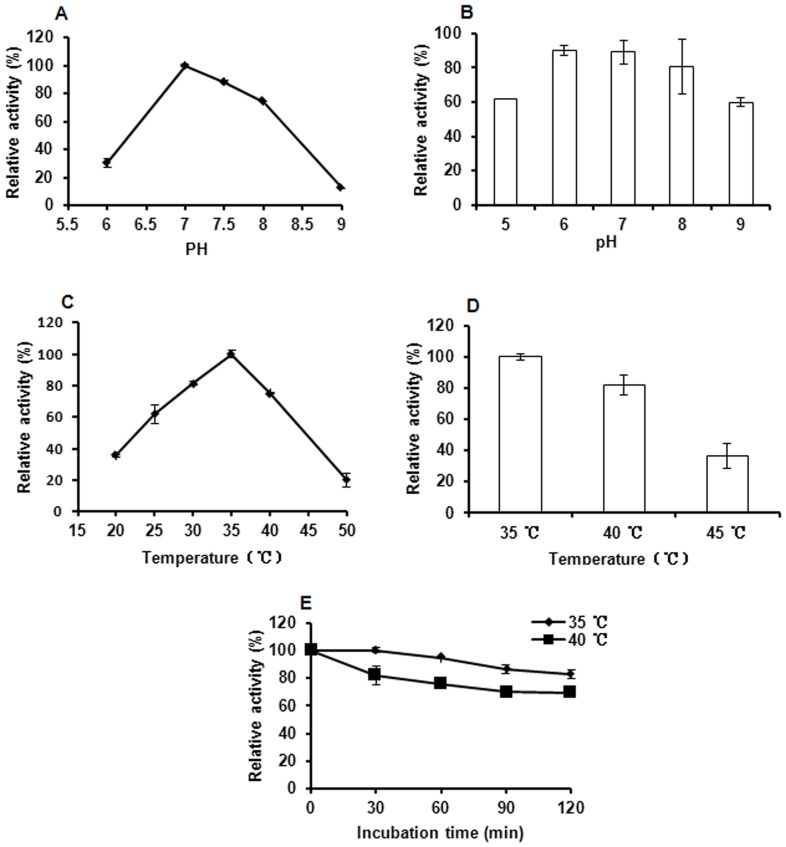
Biochemical properties of MANB. **A.** Effect of pH on the MANB activity. **B.** The pH stability of MANB. **C.** Effect of temperature on the MANB activity. **D.** The temperature stability of MANB. **E.** The enzymatic thermostability. The maximum value was considered to be 100%. The values presented correspond to the mean values of at least three replicates.

The properties of the recombinant MANB expressed by *E. coli* were determined at different pH (6–9) and temperature (20–50°C) values. The recombinant MANB showed maximal activity at pH 7.0, and more than 70% of its optimal activity was maintained at pH 7.0–8.5 (Figure 5A). Similarly, the optimal pH values of β-mannosidases produced by *Streptomyces sp.*, *Cellulomonas fimi* and *Thermobifida fusca* are 7.0–7.2 [5], [8], [17]. However, different optimum pH values exist in some strains such as *Thermoascus aurantiacus* (optimum pH: 2.5–3.0) and *Aspergillus niger* ATCC-46890 (optimum pH: 2.4–5.0) [10], [15]. In addition, the recombinant MANB from *E. coli* pET30a-MANB had considerable pH stability and over 59% of the maximal activity remained after incubation at 35°C and pH 5.0–9.0 for 30 min (Figure 5B), which corresponded with that of β-mannosidase from *E. coli* pET-man2S27 [5].

The temperature optima for the MANB produced by *E. coli* pET30a-MANB was 35°C (Figure 5C), which was lower than that reported previously [5], [8], [17]. This is probably attributed to the protein structure. Unlike other β-mannosidases, the MANB protein has no disulfide bridges or N-terminal thermostabilizing domain [17]. The recombinant MANB was relatively stable at 35°C and 40°C, and it retained over 80% of the total activity at these temperatures for 2 h (Figure 5D–E). However, it was rapidly inactivated at 45°C for 30 min (Figure 5D).

### Effect of metal ions on MANB activity

**Figure 6:**
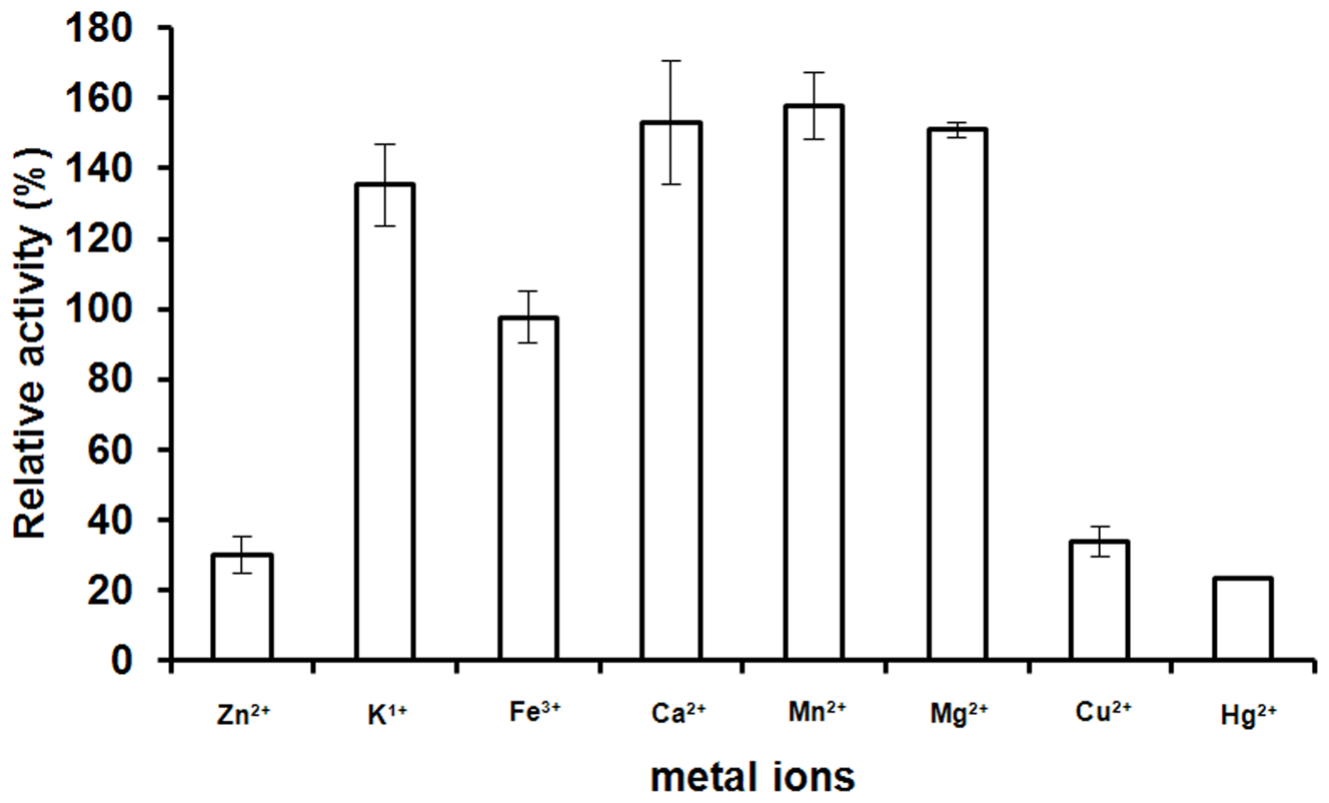
Effect of metal ions on the enzyme activity of MANB. The maximum value was considered to be 100%. The values presented correspond to the mean values of at least three replicates.

The effect of metal ions on the recombinant MANB activity was also detected (Figure 6). The effect of Fe^3+^ on the MANB activity was negligible, while K^+^, Ca^2+^, Mn^2+^ and Mg^2+^ significantly enhanced the MANB activity (135–157%). However, its activity was markedly inhibited by Zn^2+^, Cu^2+^ and Hg^2+^. Similar results have been reported for *Streptomyces sp.* S27 β-mannosidases activity which was increased by Mg and inhibited by Zn^2+^, Cu^2+^ and Hg^2+^ [5].

## Conclusions

The *MANB* gene was successfully cloned from *P. polymyxa* A-8 and expressed in *E. coli* BL21. The recombinant MANB produced in *E. coli* pET30a-MANB had considerable pH and temperature stability. Moreover, the development of more powerful expression systems such as *Picha pastoris* will be considered in our laboratory for further research.
